# Radiographic and Demographic Factors Associated with Syndesmotic Screw Breakage in Ankle Fractures

**DOI:** 10.3390/jcm15072647

**Published:** 2026-03-31

**Authors:** Emre Kocazeybek, Mehmet Ekinci, Salih Magi, Murat Altunsoy, Kubilay Yolaçan, Murat Yılmaz, Mehmet Ersin

**Affiliations:** 1 Department of Orthopaedics and Traumatology, Haseki Training and Research Hospital, University of Health Sciences, Istanbul 54600, Turkey; murataltinsoy27@gmail.com (M.A.); kubilay.yolacan@gmail.com (K.Y.); myilmaz197434@gmail.com (M.Y.); drmehmetersin@gmail.com (M.E.); 2 Department of Orthopaedics and Traumatology, Marmara University Pendik Research and Training Hospital, Istanbul 34899, Turkey; dr.ekincimehmet@gmail.com (M.E.); salihmagi73@gmail.com (S.M.)

**Keywords:** syndesmotic screw, screw breakage, ankle fracture, Weber C fracture

## Abstract

**Background:** Syndesmotic screw breakage is a well-recognized mechanical complication following ankle fracture fixation. Although several studies have investigated patient-related and technical factors associated with screw breakage, the temporal pattern of screw failure and implant survival remains less clearly defined. Therefore, this study aimed to evaluate one-year syndesmotic screw survival using time-to-event analysis and to identify factors associated with screw breakage. **Materials and Methods:** A total of 132 patients with unstable AO-Weber 44-B/C ankle fractures treated with syndesmotic screw fixation were retrospectively analyzed. Patients were followed for a minimum of 12 months or until screw breakage occurred. Screw survival was evaluated using Kaplan–Meier analysis and Cox proportional hazards regression was performed to identify factors associated with screw breakage. Demographic variables, fracture type, and screw-related parameters were analyzed. Receiver operating characteristic (ROC) analysis was used to assess the discriminative ability of age. **Results:** Screw breakage occurred in 31 patients (23.5%) during follow-up. Kaplan–Meier analysis demonstrated significantly lower screw survival in Weber C fractures compared with Weber B fractures (log-rank *p* < 0.001). Cox regression analysis identified younger age (HR: 0.965, 95% CI: 0.937–0.993, *p* = 0.016) and Weber C fracture type (HR: 1.811, 95% CI: 1.260–2.602, *p* = 0.001) as independent predictors of screw breakage. ROC analysis showed that age had moderate discriminative ability (AUC: 0.719, 95% CI: 0.612–0.816), with a cut-off value of 35.5 years. **Conclusions:** Younger age and Weber C fracture type are associated with an increased risk of syndesmotic screw breakage and Weber C fractures also demonstrating reduced screw survival. These findings may assist in patient counseling; however, the clinical implications of screw breakage remain uncertain.

## 1. Introduction

Ankle fractures are among the most common trauma referrals to orthopedic emergency departments [1]. Approximately 15–25% of ankle fractures are accompanied by syndesmotic injuries that require operative treatment [2,3].

In the management of syndesmotic injuries, cortical screws and suture-button devices are the most commonly used fixation methods. Metallic screws have historically been the standard method of fixation; however, the use of suture-button devices has become increasingly popular in recent years [4]. Although cortical screws provide stable fixation that facilitates healing of the syndesmotic ligaments, several complications have been reported including malreduction, restriction of ankle motion and screw breakage under mechanical load [5]. Consequently, the management of syndesmotic screws after fracture healing remains controversial. While some authors advocate routine removal of metallic screws after weight-bearing is permitted, others recommend leaving the screws in place unless complications occur [6]. Therefore, no definitive consensus has been reached in the literature regarding the optimal management of syndesmotic injuries [4,7].

From a clinical perspective, understanding the factors associated with syndesmotic screw breakage may help surgeons better inform patients about the expected postoperative course. Although the present study does not evaluate clinical outcomes, knowledge regarding the timing and frequency of screw breakage may contribute to discussions regarding implant retention or routine screw removal, which remains a debated issue in the literature.

Several studies have investigated factors associated with syndesmotic screw breakage and have attempted to identify independent predictors of mechanical failure. Studies by Atilla and Vander Maten et al. [2,3] evaluated potential risk factors such as patient age, fracture characteristics and screw positioning, suggesting that both demographic and radiographic parameters may influence the likelihood of screw breakage.

However, most previous studies have evaluated screw breakage as a binary outcome, and the time-dependent nature of screw failure and implant survival has not been adequately investigated. Consequently, the temporal pattern of screw breakage and its relationship with patient- and fracture-related factors remain incompletely understood.

Therefore, the aim of this study was to determine the one-year survival of syndesmotic screws in patients with ankle fractures and to identify factors associated with screw breakage using time-to-event analysis. We hypothesized that younger age and more unstable fracture patterns, particularly Weber C fractures, would be associated with decreased screw survival and an increased risk of screw breakage.

## 2. Materials and Methods

### 2.1. Patients and Study Design

Patients who underwent surgical treatment for ankle fractures with syndesmotic screw fixation between February 2017 and December 2024 were retrospectively reviewed. Demographic, clinical, and radiographic data were obtained from hospital records and the institutional imaging database. To ensure reliable evaluation of screw integrity, only patients with a minimum follow-up of 12 months or those who developed screw breakage during the follow-up period were included. One-year screw survival was defined as the primary endpoint of the study. The time to screw breakage was determined based on routine postoperative follow-up radiographs and recorded in months. Patients who experienced screw breakage were followed until the time of the event. Patients without screw breakage were followed for at least 12 months and were censored at their last follow-up visit in the survival analysis.

The STROBE (STrengthening the Reporting of OBservational studies in Epidemiology.) guidelines were followed when reporting study results [8] (Table S1). The institutional review board approved the study protocol, and this study was carried out in accordance with the ethical standards of the Declaration of Helsinki (IRB approval number: 120–2025).

All consecutive patients with AO 44-B/C fractures with syndesmotic injury who were treated with syndesmosis screw (SS) fixation were included. According to our institutional clinical protocol, syndesmotic injuries are stabilized via single or double 3.5 mm cortical screws with three or four cortices of fixation. SS are not routinely removed unless there are clear clinical indications, such as infection or wound dehiscence.

The inclusion criteria for this study were as follows: (1) patients who underwent surgery for isolated AO 44-B/C ankle fractures with syndesmotic injuries, (2) patients with adequate preoperative and postoperative ankle anteroposterior (AP), lateral and mortise radiographs and (3) patients with a minimum follow-up of 12 months or those who developed screw breakage during the follow-up period. The exclusion criteria were (1) patients treated with only suture buttons or hybrid (screw and suture button) fixation for syndesmotic stabilization, (2) a history of trauma or prior surgery on the affected ankle, (3) radiographs unsuitable for accurate measurements, (4) discontinuous follow-up, (5) the absence of adequate preoperative or postoperative radiographs and (6) follow-up shorter than 12 months without evidence of screw breakage.

### 2.2. Surgical Technique

All interventions were performed under regional or general anesthesia with a thigh tourniquet applied in the supine or lateral decubitus position. Depending on the fracture classification and whether fixation of the posterior malleolus was planned, patients were positioned either in the lateral decubitus position (for posterior malleolar fixation) or in the supine position (if the posterior fragment was absent or deemed not to require fixation). Fixation was initiated with either the posterior malleolus or the lateral malleolus (LM) on the basis of the surgeon’s preference. For LM, a distal anatomical fibular plate was used, whereas a one-third tubular plate was preferred in cases of suprasyndesmotic fractures. Posterior malleolar fractures were treated with either 3.5 mm cannulated cortical screws or a buttress plate technique. The medial malleolar fractures were subsequently fixed according to the size and morphology of the fracture fragment, using either screws, cerclage compression wiring or a buttress plate as appropriate.

After the fixation of all syndesmotic components, the stability of the syndesmotic integrity was reassessed under fluoroscopy. It was evaluated via both the external rotation stress test and the hook test. In cases demonstrating syndesmotic instability, a single or double 3.5 mm cortical SS was placed through either the designated hole in the lateral fibular plate or outside the plate, engaging three or four cortices, under fluoroscopic guidance. During screw placement, the ankle was held in a neutral position to prevent malreduction or rotational malalignment.

### 2.3. Postoperative Rehabilitation

All patients were initially immobilized in a short leg splint for two weeks until suture removal. Then, active-assisted ankle range-of-motion exercises were initiated and patients were followed with the use of an elastic bandage for inflammation control. Routine clinical and radiographic follow-up assessments were conducted on the 2nd and 6th weeks and the 3rd, 6th, 12th and 18th months postoperatively. Full weight-bearing was gradually permitted between eight and tenth weeks, depending on individual tolerance and radiological evidence of fracture union. Fracture union was determined based on both clinical findings including the absence of tenderness at the fracture site and painless full weight-bearing and radiographic evidence of bony bridging across at least three cortices. Return to pre-injury activity level was allowed following confirmation of fracture union.

### 2.4. Outcome Measurements

#### Clinical and Radiological Measurements

The demographic characteristics of the patients, body mass index (BMI), screw length, vertical distance of screw placement from the tibial plafond, insertion angle and the distance from the screw head to the site of screw failure were assessed. All data were retrospectively retrieved and measured using the hospital’s Picture Archiving and Communication System (PACS). The vertical distance between the screw and the tibial plafond was measured at the midpoint of the plafond on standard ankle radiographs. The angle between the screw and the tibial plafond (aSP) was measured in the coronal plane and screw direction was categorized as cranial, neutral or caudal according to this angle. Radiological assessments were performed by a senior foot and ankle specialist.

### 2.5. Statistical Analysis

Statistical analyses were performed using IBM SPSS Statistics (version 27.0; IBM Co., Armonk, NY, USA). Descriptive statistics (mean, standard deviation, frequency, percentage, minimum, maximum) were used to summarize the data. Normality was assessed using the Kolmogorov–Smirnov test. Between-group comparisons were made using Student’s *t*-test, the Mann–Whitney U test or the chi-square test, as appropriate. Implant survival was evaluated using Kaplan–Meier survival analysis, with time to screw breakage defined as the interval between surgery to the first radiographic detection of breakage. Patients who did not experience screw breakage were censored at their last follow-up. Cox proportional hazards regression was used to identify independent predictors of screw breakage, with results reported as hazard ratios (HR) and 95% confidence intervals (CI). Sensitivity, specificity and the area under the receiver operating characteristic (ROC) curve (AUC) were calculated. The optimal cut-off value was defined as the point providing the best balance between sensitivity and specificity. A *p*-value < 0.05 was considered statistically significant.

## 3. Results

**Figure 1:**
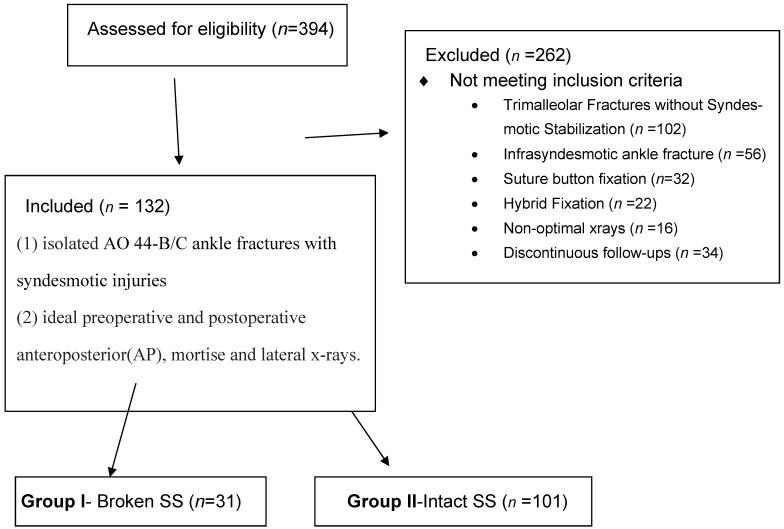
Flow diagram of the study.

During the study period, a total of 394 patients underwent surgery due to ankle fractures. Of these, 132 patients met the inclusion criteria and were included in the final analysis. Among the remaining patients, 102 had trimalleolar fracture components and underwent surgery without syndesmotic fixation, 56 patients underwent surgery for infrasyndesmotic ankle fractures, 32 patients were fixed with suture button systems alone and 22 patients were treated with hybrid fixation combining a syndesmotic screw and a suture-button device. However, the radiographic evaluation was suboptimal in 16 patients and 34 patients were excluded from the final analysis because they were lost to follow-up (Figure 1).

The demographic and clinical characteristics of the patients are summarized in Table 1. A total of 132 patients were included, with 31 in the broken syndesmotic screw (SS) group and 101 in the intact SS group. The mean age was 32.13 ± 13.22 years (range, 15–69) in the broken SS group and 44.24 ± 16.28 years (range, 17–79) in the intact SS group (*p* < 0.001). In the broken SS group, 8 patients (%25.8) were female, and 23 (%74.2) were male, whereas in the intact SS group, 41 patients (%40.59) were female, and 60 (%59.41) were male. The mean BMI was 26.8 ± 5.45 kg/m^2^ (range, 21–34.6) in the broken SS group and 27.1 ± 4.85 kg/m^2^ (range, 20.8–35.9) in the intact SS group (*p* = 0.35). According to the AO classification, the fracture type distribution was significantly different between the groups (*p* = 0.0031); in the broken SS group: 44-B1 (*n* = 5), 44-B2 (*n* = 7), 44-B3 (*n* = 3), 44-C1 (*n* = 10), and 44-C2 (*n* = 6); and in the intact SS group: 44-B1 (*n* = 36), 44-B2 (*n* = 42), 44-B3 (*n* = 11), 44-C1 (*n* = 7), and 44-C2 (*n* = 5).

**Figure 2:**
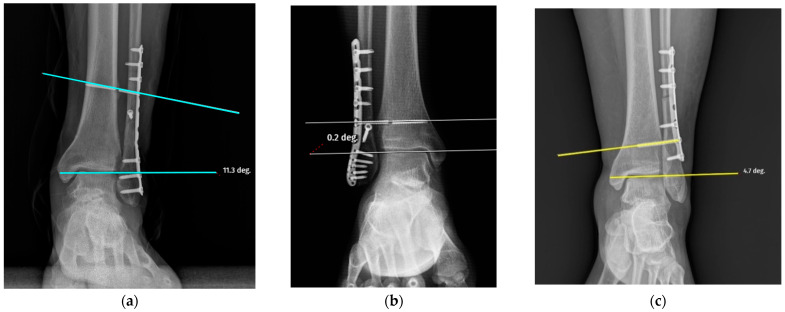
Schematic representation of screw positioning relative to tibial plafond: (**a**) the cranial position (turquoise line); (**b**) the neutral position (white line); (**c**) caudal position (yellow line).

**Figure 3:**
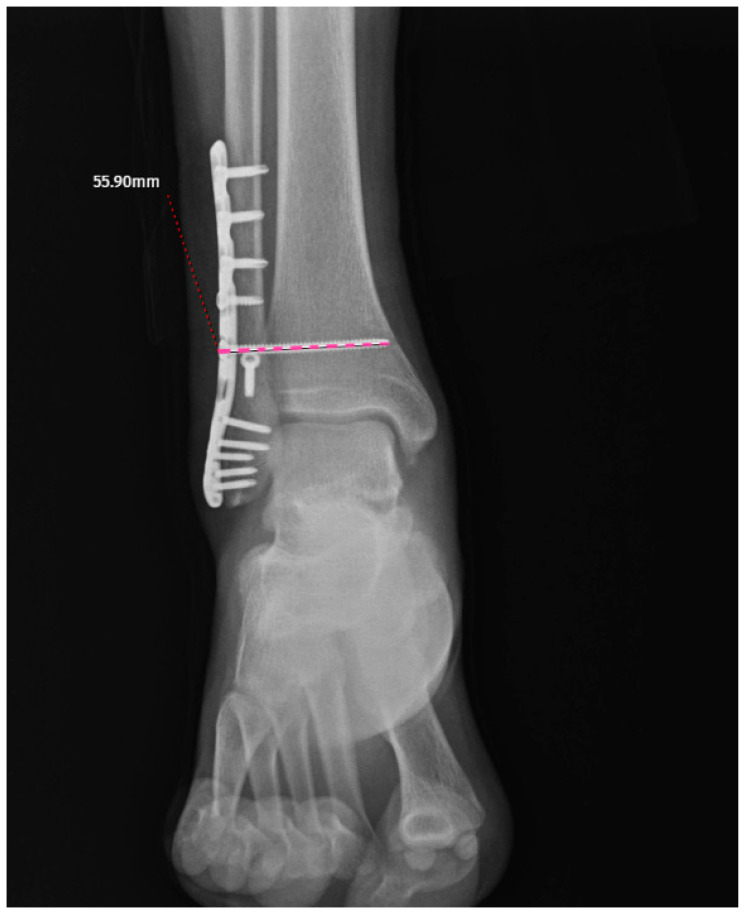
Screw length (dotted purple line).

**Figure 4:**
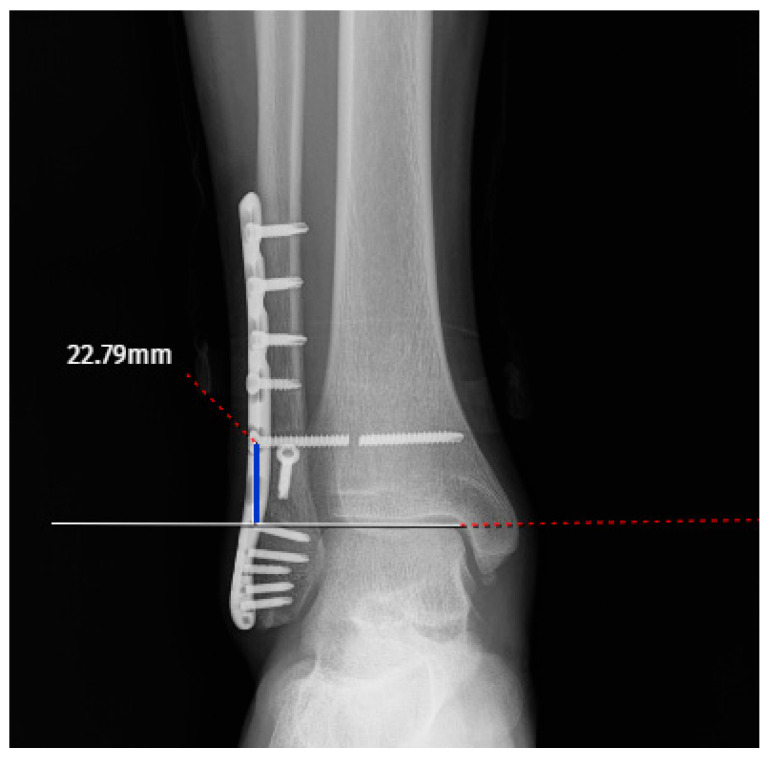
Screw distance to the tibio-talar joint (blue line).

**Figure 5:**
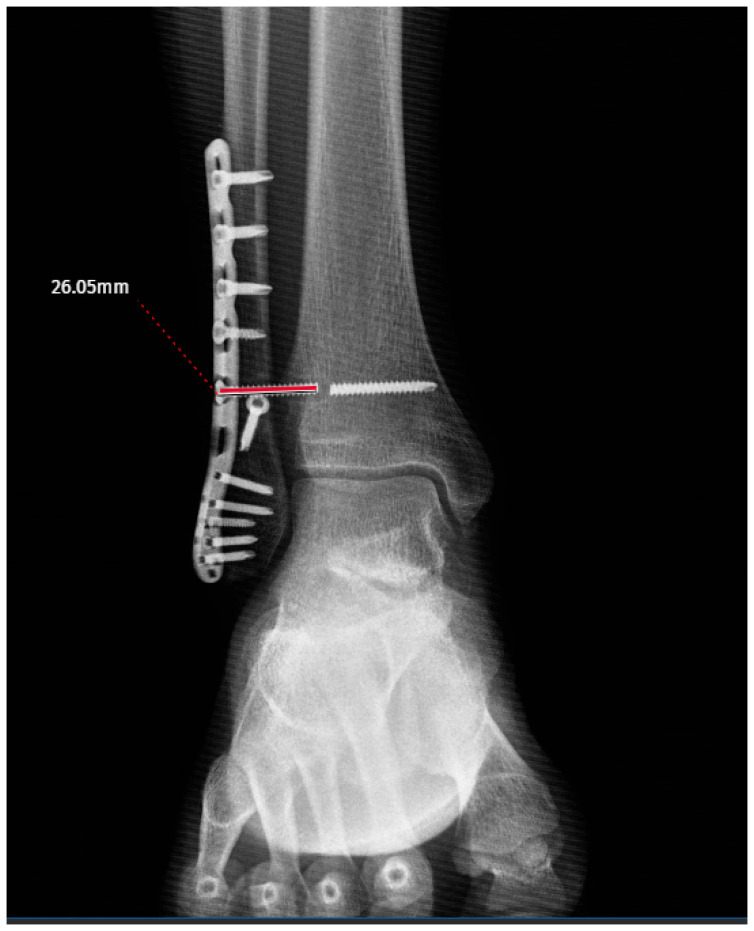
Measurement of the distance between the screw head and the breakage site (red line).

The screw-related parameters of the groups are summarized in Table 2. The screw direction showed a borderline association with breakage (cranial: 10 vs. 43; neutral: 15 vs. 51; caudal: 6 vs. 7; *p* = 0.052) (Figure 2a–c). The mean screw length was slightly greater in the broken group (53.13 ± 9.65 mm) than in the intact group (49.96 ± 7.50 mm), although this difference did not reach statistical significance (*p* = 0.106) (Figure 3). Other parameters, including screw angulation (aTP: 2.93 ± 4.00° vs. 2.76 ± 3.05°, *p* = 0.942), distance from the tibial plafond to the screw (dTP: 27.87 ± 12.32 mm vs. 26.06 ± 6.99 mm, *p* = 0.304) (Figure 4) and number of cortices engaged (three cortices: 21 vs. 66, four cortices: 10 vs. 35, *p* = 0.806), did not differ significantly between the groups. Screw breakage predominantly occurred within the first period after full weightbearing (4–6 months: 51.6%; 6–8 months: 35.5%). The distance from the screw head to the breakage point (dSH) in the broken group was 15.50 ± 8.21 mm (range: 2.98–29.20) (Figure 5). Overall, although the differences in the screw-related parameters did not reach statistical significance, the trends observed in the screw direction may indicate potential associations with breakage.

The effect of the number of syndesmotic screws on screw breakage was analysed by comparing single-screw and double-screw constructs. Screw breakage occurred in 27 patients with a single screw (22.1%) and in 4 patients with two screws (40%). Although the breakage rate was numerically greater in the two-screw group, this difference was not statistically significant (*p* = 0.37). These findings indicate that, in our cohort, the number of screws did not demonstrate a significant association with screw breakage. A detailed comparison is presented in Table 3.

**Figure 6:**
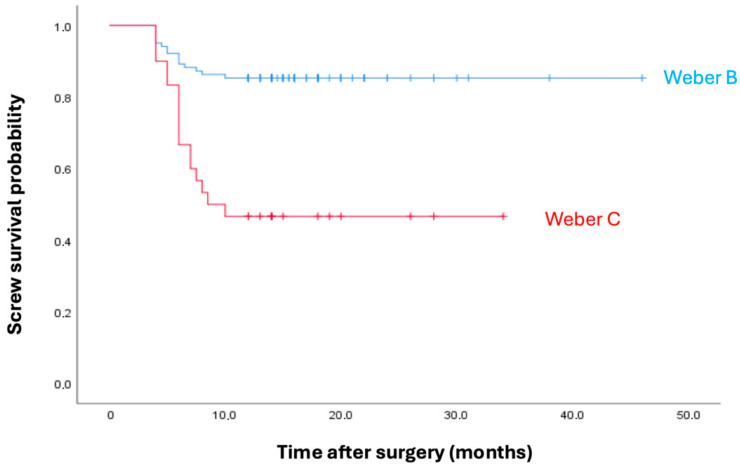
Kaplan–Meier survival curves showing screw survival according to fracture type. Weber C fractures demonstrated significantly lower screw survival compared with Weber B fractures (log-rank test, *p* < 0.001).

A total of 132 patients were included in the survival analysis. During the follow-up period, syndesmotic screw breakage occurred in 31 patients (23.5%), while 101 patients (76.5%) were censored due to the absence of screw breakage at the last follow-up. Kaplan–Meier survival analysis demonstrated a significant difference in screw survival between fracture types. Screw breakage occurred in 15 of 102 patients with Weber B fractures and in 16 of 30 patients with Weber C fractures. The one-year screw survival rate was higher in Weber B fractures compared with Weber C fractures. Kaplan–Meier analysis revealed significantly lower screw survival in Weber C fractures than in Weber B fractures (χ^2^ = 19.94, *p* < 0.001) (Figure 6). Cox proportional hazards regression analysis was performed to identify independent predictors of syndesmotic screw breakage. In the initial model including age, BMI, sex, and fracture type, age (HR: 0.965, 95% CI: 0.937–0.993, *p* = 0.016) and Weber C fracture type (HR: 1.811, 95% CI: 1.260–2.602, *p* = 0.001) were significantly associated with screw breakage, whereas BMI and sex were not significant predictors. After backward stepwise selection, age and fracture type remained independent predictors of screw breakage. Increasing age was associated with a decreased risk of screw breakage (HR: 0.959, 95% CI: 0.932–0.986, *p* = 0.003). In contrast, Weber C fractures were associated with a significantly higher risk of screw breakage compared with Weber B fractures (HR: 1.881, 95% CI: 1.317–2.686, *p* = 0.001) (Table 4). These findings were consistent with the Kaplan–Meier survival analysis, which also demonstrated significantly lower screw survival in Weber C fractures.

**Figure 7:**
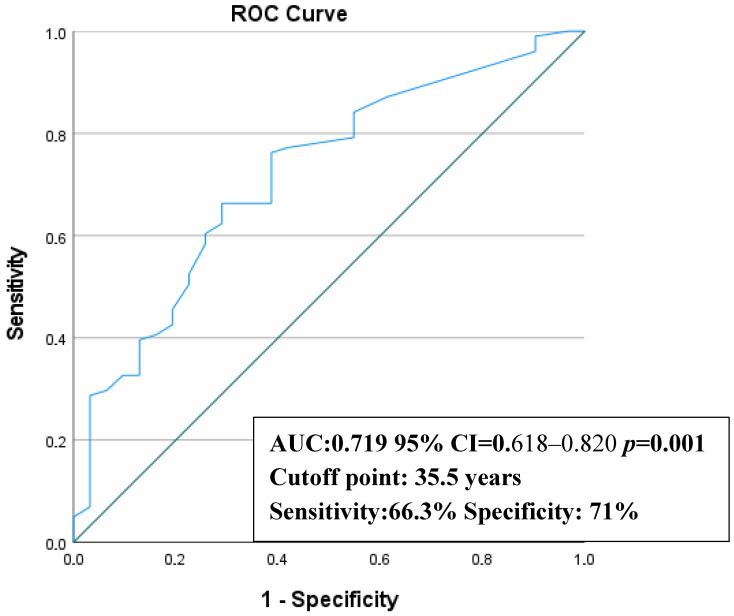
Receiver Operating Characteristics Curve (ROC).

ROC curve analysis, performed to further evaluate the predictive value of age identified in Cox regression analysis, demonstrated that age had a moderate discriminative ability for predicting screw breakage (AUC = 0.719, 95% CI: 0.612–0.816). The optimal cut-off value was 35.5 years, with a sensitivity of 66.3% and a specificity of 71.0% (Figure 7).

## 4. Discussion

In the present study, it is aimed to evaluate the one-year survival of syndesmotic screws and to identify factors associated with screw breakage using time-to-event analysis. The main findings of this study demonstrate that screw breakage occurred in approximately one-quarter of patients, while younger age and Weber C fracture type were independently associated with an increased risk of screw failure.

In addition, Weber C fractures were associated with reduced screw survival over time. These findings directly address the study objective and highlight the role of both patient-related and fracture-related factors in the time-dependent behavior of syndesmotic fixation.

The first independent predictor identified in the present study was age. Increasing age was associated with a reduced risk of screw failure. This finding is consistent with previous studies suggesting that younger patients may place greater mechanical stress on the fixation construct because of higher activity levels during rehabilitation. Age has been reported as an important factor associated with syndesmotic screw breakage in several studies. Boyle et al. [9], in a prospective randomized trial, observed that patients with broken syndesmotic screws tended to be younger at the one-year follow-up; however, the limited sample size may have precluded the detection of a statistically significant association. Similarly, Behery et al. [10] reported that patients with broken screws were significantly younger than those with intact screws. In addition, Hamid et al. [11] found that patients with screw breakage had a lower mean age compared with those in whom the screws were retained or removed and suggested that this may be related to higher activity levels and more intensive rehabilitation in younger individuals. Nevertheless, none of these studies defined a specific threshold value for age, which may limit the clinical applicability of their findings. In contrast, Atilla et al. [2] identified age as an independent risk factor and reported a cut-off value of 36 years. Consistent with these findings, age was also found to be an independent predictor of screw breakage in our study, with a statistically derived cut-off value of 35.5 years, which may be specific to the characteristics of our study population.

The second independent predictor significantly associated with syndesmotic screw breakage was fracture type. The number of studies specifically evaluating the relationship between screw breakage and the Weber classification is limited. Bell et al. [12] reported that Weber C fractures are associated with a higher likelihood of screw breakage and osteolysis, particularly when screws are retained after weight-bearing. Similarly, Ortiz et al. [13] observed a high rate of screw breakage in Weber C fractures, although they did not recommend routine screw removal due to the lack of significant differences in clinical outcomes. In addition, van den Bekerom et al. [5], in a large cohort reported that screw breakage occurred more frequently in Weber C fractures. These findings may be explained by the biomechanical characteristics of these injuries. Weber C fractures are typically associated with more extensive disruption of the syndesmotic complex and greater rotational instability of the ankle mortise. Chen et al. [14] demonstrated that the injury mechanism affects the stability of syndesmotic fixation, with more unstable patterns resulting in increased mechanical stress across the fixation construct. In this context, increased instability may lead to higher cyclic loading across the syndesmotic screw during postoperative weight-bearing, thereby predisposing the implant to fatigue failure. In line with these findings, fracture type was identified as an independent predictor of screw breakage, with Weber C fractures associated with a higher risk of breakage and lower screw survival compared with Weber B fractures.

Beyond age and fracture type, several demographic variables have been examined in the literature for their potential association with syndesmotic screw breakage. Previous studies by Behery and Walker reported a higher incidence of screw breakage in male patients, suggesting that sex may influence implant behavior and loading characteristics [10,15]. However, in our cohort, sex was not found to be significantly associated with syndesmotic screw breakage, as no statistically significant difference was observed between male and female patients.

The literature presents conflicting evidence regarding the association between BMI and syndesmotic screw breakage. Several studies have reported that BMI is not a significant risk factor [2,9,10], whereas Vander Maten et al. [3] found an association between higher BMI and intraosseous screw breakage. In line with most previous reports, BMI was not identified as an independent predictor of screw breakage in our cohort.

Recent attention has focused not only on demographic predictors but also on screw-related mechanical factors, particularly coronal-plane orientation, the degree of cortical engagement and the screw’s distance from the tibial plafond. The orientation of the screw may affect the distribution of forces across the distal tibiofibular joint, while screw length determines the number of cortices engaged and may influence construct stability. These factors may alter the mechanical stress experienced by the screw during ankle motion and weight-bearing, potentially contributing to implant fatigue and screw breakage. Previous studies have also examined the influence of screw orientation and placement characteristics in the coronal plane on the risk of syndesmotic screw breakage, yet the results remain inconclusive. Vander Maten et al. demonstrated that screws positioned more than 20 mm above the tibial plafond were associated with higher breakage rates; however, screw angulation in the coronal plane was not significantly correlated with failure [3,16]. In a subsequent investigation by the same group, intraosseous fibular screw breakages were associated with greater screw angulation and increased patient age, whereas other factors, including screw height, were not significantly related to the location of failure. These findings suggest that an altered screw trajectory may contribute to mechanical stress within the fibula, although the evidence remains inconsistent. In contrast, Atila et al. reported no significant association between coronal plane screw characteristics and breakage, thereby questioning the clinical relevance of such parameters [2]. Similarly, our study demonstrated that screw-related parameters, including the number of cortices engaged, screw length and distance from the tibial plafond in the coronal plane were not significantly associated with the occurrence of syndesmotic screw breakage. Collectively, these findings suggest that although such mechanical parameters may theoretically influence implant behavior, their contribution appears limited when compared with patient-related and fracture-specific characteristics.

The location of syndesmotic screw breakage relative to the fibular cortex has also been explored in the literature. Stenquist [17] et al. demonstrated that controlled screw failure commonly occurs at 14–17 mm, corresponding to the fibular thickness, whereas Atilla et al. [2] identified a mean breakage distance of 19.3 mm from the screw head, thereby indicating a predominance of intraosseous failure. Consistent with these reports, our results revealed a mean breakage distance of 15.50 ± 8.21 mm from the screw head, further supporting the notion that screw failure is not a random phenomenon but rather follows characteristic biomechanical patterns. Vander Maten et al. [3] expanded on this concept by demonstrating that intraosseous breakages become increasingly frequent with advancing age, suggesting an interaction between biological and mechanical determinants.

In our series, most syndesmotic screw breakages clustered between the 4th and 8th postoperative months, after which the incidence markedly declined. This pattern is consistent with previous reports, which similarly demonstrated that screws retained beyond the early postoperative period have a substantially increased risk of failure [18,19]. These findings underscore the time-dependent nature of syndesmotic screw breakage, with the highest vulnerability occurring within the midterm follow-up window.

In the present study, routine removal of syndesmotic screws was not part of the standard postoperative protocol, and clinical outcome measures were not compared between patients with broken and intact screws. Accordingly, no conclusions regarding the clinical consequences of screw breakage can be drawn from our data. However, contemporary literature suggests that routine syndesmotic screw removal is not supported by high-level evidence and should be considered on an individualized basis rather than performed systematically [20,21]. From this perspective, our findings may contribute to patient counseling, particularly in informing younger patients and those with more unstable fracture patterns about the increased likelihood of screw breakage, while its clinical implications remain uncertain.

Several limitations of the present study should be acknowledged. First, the retrospective design inherently limits the ability to establish causal relationships. Second, surgical procedures were performed by multiple surgeons during the study period and variations in individual surgical preferences may have introduced selection bias. Third, radiographic assessments were performed by a single senior foot and ankle specialist; therefore, inter- and intraobserver reliability could not be evaluated. This represents an important limitation of the study, as measurement variability could not be assessed.

Fourth, the relatively small sample size, particularly in the screw breakage subgroup, and the limited number of female patients may have reduced the statistical power of the study and limited the ability to detect potential associations, especially with respect to sex. Therefore, the absence of a statistically significant relationship should be interpreted with caution. Additionally, information regarding patients’ postoperative activity levels and occupational demands was not available due to the retrospective design of the study. Such factors may influence the mechanical loading of syndesmotic screws and potentially affect the risk of screw breakage. Data regarding patient compliance with postoperative rehabilitation protocols and weight-bearing restrictions were also not consistently documented, which may have influenced the outcomes.

Finally, the absence of clinical and functional outcome measures precludes any conclusions regarding the clinical impact of syndesmotic screw breakage. Despite these limitations, the study provides descriptive data on patient- and fracture-related factors associated with syndesmotic screw breakage, which may serve as a basis for hypothesis generation and future prospective investigations.

## 5. Conclusions

In conclusion, syndesmotic screw breakage was more frequently observed in younger patients and in Weber C fracture patterns, both of which were identified as independent predictors of screw breakage. In addition, Weber C fractures were associated with reduced screw survival. These findings suggest that fracture characteristics and patient-related factors influence the mechanical behavior of syndesmotic fixation. This may be explained by increased mechanical loading in younger patients and greater syndesmotic instability in Weber C fractures, both of which may contribute to higher stress on the fixation construct. The primary value of these findings lies in informing preoperative patient counseling and in setting appropriate expectations regarding the likelihood of screw breakage. However, as clinical outcomes were not evaluated in this study, no definitive conclusions can be drawn regarding the clinical significance of screw breakage and further prospective studies are required to clarify its clinical impact.

## Figures and Tables

**Table 1:** Demographic characteristics of the patients.

Variable		Group 1 (Broken SS) (*n* = 31)	Group 2 Intact SS (*n* = 101)	
Age (year)	Mean ± SD	32.13 ± 13.22	44.24 ± 16.28	*p* < 0.001
	Min–Max	(15–69)	(17–79)	
Sex	Female	8 (%25.8)	41 (%40.59)	0.201
	Male	23 (%74.2)	60 (%59.41)	
BMI (kg/m^2^)	Mean ± SD	26.8 ± 5.45	27.1 ± 4.85	*p* = 0.35
	Min–Max	(21–34.6)	(20.8–35.9)	
AO Classification (*n*)				*p* = 0.0031
44-B1		5	36	
44-B2		7	42	
44-B3		3	11	
44-C1		10	7	
44-C2		6	5	

**Table 2:** Screw parameters between groups.

		Group 1 (Broken SS) (*n* = 31)	Group 2 Intact SS (*n* = 101)	
Screw Direction (*n*)				*p* = 0.052
			
Cranial		10	43	
			
Neutral		15	51	
			
Caudal		6	7	
dTP (mm)	Mean ± SD	27.87 ± 12.32	26.06 ± 6.99	*p* = 0.304
	Min–Max	(12.06–72.70)	(11.02–45.65)	
aTP (degrees)	Mean ± SD	2.93 ± 4.00	2.76 ± 3.05	*p* = 0.942
	Min–Max	(5.2–12.40)	(1.2–12.7)	
Screw Length (mm)	Mean ± SD	53.13 ± 9.65	49.96 ± 7.50	*p* = 0.106
	Min–Max	(36.18–77.25)	(29.57–68.80)	
Number of cortices (n)	Three	21	66	*p* = 0.806
	Four	10	35	
Time to breakage (mo)	Median	6		Not calculated
	(range)	(4–10)		
dSH	Mean ± SD	15.50 ± 8.21		
	Min–Max	(2.98–29.20)		

**aTP:** Angle relative to the tibial plafond. **dTP:** Distance relative to the tibial plafond. **dSH**: Breakage distance from the screw head.

**Table 3:** Comparison of Screw Breakage by Number of Screws.

Group	Single Screw (*n* = 122)	Double Screws (*n* = 10)	Total	
Broken	27	4	31	
Intact	95	6	101	
Breakage Rate	22.1%	40%	132	*p* = 0.37

**Table 4:** Cox Proportional Hazards Regression Analysis for Screw Breakage (Final Model).

Explanatory	Variable	B	SE	HR (Exp(B))	95% CI	*p*
Step 6	Age	−0.042	0.014	0.959	0.932–0.986	0.003
	Weber (B vs. C)	0.632	0.182	1.881	1.317–2.686	0.001

## Data Availability

The data presented in this study are not publicly available but are available on reasonable request from the corresponding author.

## Supplementary Materials

The following supporting information can be downloaded at: https://www.mdpi.com/article/10.3390/jcm15072647/s1, Table S1: STROBE Statement—checklist of items that should be included in reports of observational studies.

## Abbreviations

SS	Syndesmotic screw
BMI	Body mass index
ROC	Receiver operating characteristic
HR	Hazard ratio
CI	Confidence interval
AO	Arbeitsgemeinschaft für Osteosynthesefragen (Fracture Classification)
AP	Anteroposterior
LM	Lateral Malleolus
PACS	Picture Archiving and Communication System
aSP	Angle between plafond and screw
